# Nauclea officinalis extract rescues working memory deficits in adolescent maternal immune activation offspring by restoring cholinergic signaling

**DOI:** 10.3389/fcell.2026.1918425

**Published:** 2026-08-20

**Authors:** Lei An, Zhanyong Li, Yan Wang, Lin Feng, Wei Sun

**Affiliations:** 1 Department of Proctology, The First Affiliated Hospital of Guizhou University of Traditional Chinese Medicine, Guiyang, China; 2 Department of Emergency, The First Affiliated Hospital of Guizhou University of Traditional Chinese Medicine, Guiyang, China; 3 School of Life Sciences, Langfang Normal University, Langfang, China; 4 Department of Neonatology, The First Affiliated Hospital of Guizhou University of Traditional Chinese Medicine, Guiyang, China

**Keywords:** cholinergic system, maternal immune activation (MIA), Nauclea officinalis extract (NOE), prefrontal cortex, working memory

## Abstract

**Objective:**

Maternal immune activation (MIA) is a key risk factor for schizophrenia-related cognitive deficits, linked to prefrontal cholinergic dysfunction. Nauclea officinalis extract (NOE) possesses documented anti-inflammatory and neuroprotective properties, but its effects on MIA-induced cognitive and neuronal dysfunctions are unknown. This study aimed to investigate whether NOE ameliorates MIA-induced working memory deficits by modulating infralimbic medial prefrontal cortex (IL-mPFC) cholinergic signaling and neuronal activity.

**Methods:**

Using a poly (I:C)-induced MIA rat model, adolescent offspring were treated with NOE (5, 10 and 20 mg/kg). Behavioral tests, *in vivo* electrophysiology, and molecular assays were conducted.

**Results:**

NOE dose-dependently attenuated MIA-induced neuroinflammation (IL-1β, IL-6, TNF-α) in the IL-mPFC. It restored acetylcholine (ACh) levels, reduced acetylcholinesterase (AChE) activity, but did not alter M1/M3 receptor expression. NOE (20 mg/kg) significantly improved 30-s delay working memory performance, an effect blocked by the muscarinic receptor antagonist scopolamine (SCO) and mimicked by the AChE inhibitor physostigmine (PHY). Electrophysiologically, NOE rescued MIA-impaired long-term potentiation (LTP) and paired-pulse ratio (PPR), and enhanced delay-related firing of IL pyramidal neurons. These restorative effects were similarly replicated by PHY and antagonized by SCO.

**Conclusion:**

NOE alleviates MIA-induced working memory deficits by reducing neuroinflammation and, crucially, by functionally potentiating cholinergic transmission in the IL-mPFC. The findings highlight the IL cholinergic system as a critical therapeutic target and position NOE as a promising multi-target agent for cognitive impairments in neurodevelopmental disorders.

## 1 Introduction

Schizophrenia, as a severe mental disorder, exhibits a complex and diverse pathogenesis involving interactions among multiple factors such as genetics, environment, and neurodevelopment (Birnbaum and Weinberger, 2017; Newbury et al., 2022). Maternal immune activation (MIA) has attracted extensive research attention as a significant etiological factor in schizophrenia (Brown and Meyer, 2018; Openshaw et al., 2019). Epidemiological evidence indicates that infection and immune activation during pregnancy significantly increase the risk of schizophrenia in offspring (Conway and Brown, 2019; Woods et al., 2026). Animal studies have further confirmed that MIA can induce schizophrenia-like behavioral abnormalities and neuropathological alterations in offspring (Talukdar et al., 2020; Sun et al., 2022a). The pathological mechanisms induced by MIA primarily include inflammatory responses, abnormal neurodevelopment, and dysregulation of neurotransmitter systems (Sun et al., 2022a; Tang et al., 2025; Kwon et al., 2022). Specifically, concerning the cholinergic system, MIA has been shown to significantly reduce the activity of cholinergic neurons, leading to decreased acetylcholine (ACh) content and abnormally elevated acetylcholinesterase (AChE) activity (Reyes-Lagos et al., 2021; Maia et al., 2025; Vaknine Treidel et al., 2025; King and Plakke, 2024). Such cholinergic system dysfunction plays a crucial role in the cognitive impairments observed in schizophrenia, particularly in working memory, attention, and executive function (King and Plakke, 2024).

The medial prefrontal cortex (mPFC) is a core brain region regulating higher cognitive functions. Its infralimbic (IL) subregion, as a key subdivision of the mPFC, occupies a unique position in neural functional regulation (Savvateev et al., 2025). It serves not only as a critical hub connecting the cortex and the limbic system but is also closely related to the pathogenesis and progression of neuropsychiatric disorders and the maintenance of cognitive functions (Cotta Ramusino et al., 2024; Sun et al., 2018). Numerous studies have confirmed that the IL region is a core regulatory site for working memory, with its functional integrity directly determining the encoding, maintenance, and retrieval processes of working memory (Mukherjee and Caroni, 2018; Liu and Carter, 2018). The neuronal activity characteristics within the IL region are highly aligned with the core processes of working memory. Notably, delayed-related activity is considered the key neural substrate for maintaining working memory information. Research demonstrates that pyramidal neurons in the IL region exhibit specific firing rate increases or suppression during the delay period of working memory tasks (Liu and Carter, 2018). This firing pattern can accurately encode task-relevant information (e.g., stimulus type, delay duration), and its firing intensity shows a significant positive correlation with the accuracy of working memory tasks (Jun et al., 2025; Porter and Sepulveda-Orengo, 2020). Furthermore, regular-spiking (RS) pyramidal neurons within the IL region optimize the precision of delayed neural signals by modulating the excitatory-inhibitory balance, ensuring the stable progression of working memory processes (Vogel et al., 2022; Tudi et al., 2024). It is noteworthy that IL region function exhibits developmental dependency and retains task-phase-specific functional specialization into adulthood (Cruz-Sanchez et al., 2026; Zimmermann et al., 2023; Karst et al., 2020). Developmental abnormalities during the embryonic or early postnatal periods (e.g., MIA, early-life stress) can directly disrupt the structural formation and functional differentiation of the IL region, leading to persistent deficits in IL-mediated cognitive functions such as working memory (Karst et al., 2020; Awad et al., 2022; Zhao et al., 2021).

The cholinergic system is the core neurotransmitter system mediating cognitive functions and neuronal activity within the IL region (Jun et al., 2025; Luo and Kiss, 2021). Its dysfunction constitutes a significant pathological basis for cognitive impairment in various neuropsychiatric disorders (Orlando et al., 2023). Cholinergic nerve fibers extensively project from the basal forebrain (e.g., medial septal nucleus, diagonal band nucleus) to the IL region, modulating neuronal activity and synaptic plasticity in this area through the release of ACh (Martínez-Murillo and Rodrigo, 2020). Within the IL region, ACh primarily exerts its regulatory effects by acting on muscarinic (M) and nicotinic receptors. Among these, the M1 and M3 receptor subtypes are particularly critical for working memory regulation (Vijayraghavan et al., 2018; Zheng et al., 2023). M1 receptors are mainly expressed on the dendrites and somata of IL pyramidal neurons. Their activation enhances the excitability and delayed firing activity of pyramidal neurons by modulating calcium influx and protein kinase C (PKC) signaling pathways, thereby promoting the maintenance of working memory information (Vijayraghavan et al., 2018). M3 receptors are primarily involved in regulating the efficiency of presynaptic ACh release, maintaining stable ACh concentration in the synaptic cleft through negative feedback regulation to ensure the precision of cholinergic signaling (Zheng et al., 2023). Existing studies have confirmed that cholinergic hypofunction directly leads to weakened delayed neuronal activity, impaired synaptic plasticity in the IL region, and ultimately, working memory deficits (Djemil et al., 2023). For instance, in animal models of schizophrenia, decreased choline acetyltransferase (ChAT) activity and increased AChE activity in the IL region result in reduced synaptic ACh levels and downregulated M1/M3 receptor expression, which significantly suppresses delayed-related activity in pyramidal neurons (Liy et al., 2019). This pathological alteration is considered one of the core mechanisms underlying working memory impairment in these models (Tsimpili and Zoidis, 2025). Furthermore, oxidative stress and neuroinflammation can indirectly disrupt cholinergic system function in the IL region by damaging cholinergic neurons and inhibiting ACh synthesis and release, forming a vicious cycle of neuroinflammation-cholinergic dysfunction-cognitive impairment (Gamage et al., 2020; Echeverria et al., 2023). This further highlights the significant value of the IL cholinergic system as a target for cognitive function regulation.

Nauclea officinalis Pierre ex Pitard (Danmu), derived from the dried stems, branches, and bark of plants belonging to the Naucleagenus in the Rubiaceae family, is a traditional Chinese medicinal plant traditionally used for treating cold, fever, sore throat, damp-heat jaundice, abscesses, and sores (Liu et al., 2022). Modern phytochemical studies indicate that the main active constituents of Danmu are alkaloids, including strictosamide, nauclefine, and paucine. Among these, strictosamide is the most abundant and biologically significant marker component (Li et al., 2017; Zhou et al., 2021). In recent years, the neuroprotective effects of Danmu extracts have gradually become a research focus. Substantial pharmacological evidence suggests that Danmu extracts exhibit significant therapeutic potential in various models of neurological disorders, demonstrating clear prospects for application, particularly in ameliorating inflammation (Liu et al., 2022; Li et al., 2025). Regarding neuroprotective mechanisms, Danmu extracts primarily act through multiple pathways, including anti-inflammatory, antioxidant, anti-apoptotic effects, and the improvement of neural function (Li et al., 2023). In terms of anti-inflammatory action, studies have found that strictosamide from Danmu extracts significantly inhibits the expression of lipopolysaccharide (LPS)-induced pro-inflammatory cytokines such as interleukin-1β (IL-1β), interleukin-6 (IL-6) and tumor necrosis factor-α (TNF-α) and downregulates the activation of the nuclear factor-kappa B (NF-κB) signaling pathway, thereby attenuating central nervous system inflammation (Li et al., 2017; Zhai et al., 2016; Xu et al., 2022). In an LPS-induced acute lung injury mouse model, Danmu extract syrup exerted protective effects by repairing the endothelial barrier and inhibiting inflammatory infiltration. Its anti-inflammatory mechanism is closely related to the modulation of inflammatory signaling pathways (Zhai et al., 2016; Xu et al., 2022), providing important experimental evidence for its potential to improve MIA-induced central inflammation. Importantly, Danmu extracts exert a clear regulatory effect on the cholinergic system (Li et al., 2023; Liew et al., 2015), which is one of the core mechanisms underlying their cognitive-enhancing properties. Studies have confirmed that Danmu extracts significantly increase brain ChAT activity, promote ACh synthesis and release, while inhibiting AChE activity and reducing ACh degradation, thereby elevating synaptic ACh concentration (Liew et al., 2015). In Alzheimer’s disease (AD) model animals, Danmu extracts improve learning, memory, and synaptic plasticity by upregulating M1 receptor expression in the hippocampus and cortex, enhancing cholinergic signaling (Li et al., 2025). In stroke models, Danmu extracts protect cholinergic neurons in ischemic regions, attenuate cholinergic system damage, and promote neurological recovery (Kong et al., 2021). Moreover, strictosamide, as the main active component of Danmu, has also been confirmed to possess significant anti-inflammatory and analgesic activities, with mechanisms related to the regulation of inflammatory factor release (Li et al., 2017; Li et al., 2014; Omar et al., 2021), providing further component-level support for the neuroprotective effects of Danmu extracts. However, current research on Danmu extracts has primarily focused on neurodegenerative diseases such as AD and PD (Li et al., 2023; Kong et al., 2021). Whether Danmu extracts ameliorate MIA-induced schizophrenia-like cognitive deficits, particularly by regulating cholinergic system function in the IL region and restoring delayed-related neuronal activity to improve working memory remains unclear.

Based on the above research background, this study aims to investigate the effects and underlying mechanisms of the acidic 70% ethanol extract of total alkaloids from Nauclea officinalis (NOE) on cognitive memory and neural activity in MIA offspring rats. Behavioral tests (Open Field Test, Lever Press Test, Odor Delayed Non-Match-to-Sample Test) were employed to assess the effects of NOE on anxiety-like behavior, motivated behavior, and working memory capacity in MIA offspring. *In vivo* electrophysiological recording techniques were used to simultaneously monitor delayed-related activity of IL region neurons (pyramidal neurons, interneurons) during working memory tasks, clarifying the regulatory effects of NOE on IL neural activity. Combined with molecular biology assays (measurement of AChE activity, ChAT activity, and ACh content; detection of M1/M3 receptor expression) and pharmacological intervention experiments (intracerebroventricular injection of AChE inhibitor and non-selective muscarinic ACh receptor antagonist), the core mechanism through which NOE influenced on cognitive deficits in MIA offspring by modulating the IL cholinergic system was elucidated. Additionally, *ex vivo* brain slice field potential recording techniques were conducted to explore the effects of NOE on synaptic plasticity (long-term potentiation (LTP) and paired-pulse ratio (PPR)) in the IL region. This study not only provides experimental evidence for the potential application of Danmu extracts in treating neuropsychiatric disorders like schizophrenia but also aims to clarify the feasibility of targeting the IL cholinergic system for treating MIA-related cognitive impairments, offering novel insights and strategies for the precise treatment of cognitive deficits in schizophrenia.

## 2 Experimental procedure

### 2.1 Experimental animals and MIA model establishment

Sprague-Dawley (SD) rats were used in this study. All animals were housed under controlled conditions: temperature (22 ± 2) °C, relative humidity (50 ± 10)%, with a 12-h light/dark cycle, and provided with *ad libitum* access to food and water. All animal procedures were performed in accordance with the regulations of the Care and Use of Animals Committee of Langfang Normal University (SCXK-2019–0010).

The MIA model was established using the polyinosinic-polycytidylic acid [poly (I:C)] induction method. On gestational day (GD) 15, pregnant dams received a single intravenous tail vein injection of poly (I:C) (4 mg/kg, InVivoGen). Control dams received an equivalent volume of saline. GD 15 was selected for immune activation based on the critical time window for cortical neurogenesis in rats, during which the developing brain is highly sensitive to external insults. Poly (I:C), a synthetic double-stranded RNA analog mimicking viral infection, induces a robust immune response by activating Toll-like receptor 3 (TLR3), leading to the production of pro-inflammatory cytokines including IL-1β, IL-6, and TNF-α.

Only male offspring were utilized in the present study. Given that IL-mPFC maturation and MIA-induced inflammatory sequelae exhibit pronounced sex-biased trajectories during the peri-adolescent period (Sal-Sarria et al., 2024; Meehan et al., 2016; Harbi and Mouihate, 2025), restricting the cohort to males allowed us to isolate NOE’s primary cholinergic-pharmacological axis without the variance introduced by gonadal hormone fluctuations. Additionally, the peri-adolescent interval was deliberately chosen because it parallels the human schizophrenia prodromal peak, a stage during which IL-mPFC circuit maturation and delay-dependent working memory undergo their most dynamic transitions (Dienel et al., 2022; Mukherjee et al., 2019; Kirschmann et al., 2019; Caballero et al., 2016).

### 2.2 MIA offspring model validation

At weaning (postnatal day 21, PND21), 5–8 offspring rats were randomly selected from each group. Following anesthesia with isoflurane (5% induction, 3% maintenance), rats were decapitated, and the brain was rapidly removed. IL-mPFC tissue was dissected, weighed, and placed in ice-cold saline. Tissue was homogenized in RIPA lysis buffer containing protease inhibitors at a weight-to-volume ratio of 1:10. Homogenates were centrifuged at 12,000 rpm for 15 min at 4 °C, and the supernatant was collected. The levels of key pro-inflammatory cytokines, including IL-1β (R&D Systems, catNo.RLB00), IL-6 (R&D Systems, catNo.R6000B), TNF-α (R&D Systems, catNo.RTA00), in the supernatant were measured using commercially available ELISA kits according to the manufacturer’s instructions with optimized key parameters: standards and samples were added to antibody-precoated wells and incubated precisely at 37 °C for 60 min. Wells were washed 5 times with TBST buffer (3 min each). Biotinylated secondary antibody was added and incubated at 37 °C for 30 min, followed by another 5 TBST washes. Horseradish peroxidase (HRP)-labeled streptavidin was added and incubated at 37 °C for 15 min. Finally, substrate solution was added and incubated at 37 °C for 15 min in the dark. The reaction was stopped, and the absorbance (OD value) of each well was measured at 450 nm using a micro-plate reader. Cytokine concentrations were calculated based on the standard curve.

### 2.3 Drug treatment and group assignment

The whole herb of Nauclea officinalis, supplied by Shaanxi Snote Biotechnology Co., Ltd., was morphologically identified by Professor Zhang (Life Science Department, Langfang Normal University). A voucher specimen for this material, numbered 20232263, is preserved in the Life Science Museum of Langfang Normal University. The acidic 70% ethanol extract of total alkaloids from Nauclea officinalis (NOE) was prepared using heat reflux extraction. Dried stems, branches, bark, and roots of Nauclea officinalis were pulverized. The powder was mixed with 70% ethanol at a solid-to-liquid ratio of 1:16 and subjected to reflux extraction at 80 °C twice, each for 40 min. The combined extracts were concentrated under reduced pressure. The pH was adjusted to 4.0–4.5 with dilute hydrochloric acid to obtain the acidic extract. The content of strictosamide in the extract was determined by High-Performance Liquid Chromatography (HPLC) as a quality control indicator. After calculation, the content of strictosamide in the acidic 70% ethanol extract of total alkaloids from *Nauclea officinalis* was required to be not less than 1.5% (w/w) of the dry weight of the extract, with the relative standard deviation of strictosamide content among different batches controlled within ≤5.0%. This standard was established based on relevant phytochemical reports (Wall et al., 2001; Boix-Trelis et al., 2007) and pre-experimental data, which confirmed that NOE meeting this criterion exhibited stable anti-inflammatory.

Based on preliminary experimental results and well-established dose paradigms for plant alkaloid extracts targeting neuroinflammation and cholinergic function in adolescent rodent models (Wall et al., 2001; Boix-Trelis et al., 2007), three gradient NOE doses (5, 10, and 20 mg/kg) were selected. This dosage range aligns with published preclinical data for NOE and its major active constituent strictosamide; doses within 5–20 mg/kg achieve effective central exposure without observable systemic toxicity in adolescent rats, and support stable blood-brain barrier penetration to exert anti-inflammatory and cholinergic regulatory actions (Wall et al., 2001; Boix-Trelis et al., 2007). The three-tiered dosing scheme was designed to characterize dose-dependent responses, enabling discrimination of subtherapeutic marginal effects, moderate functional improvement, and maximal therapeutic efficacy against MIA-induced neuroinflammation, cholinergic dysfunction and working memory deficits.

The administration volume was calculated as 10 μL/g body weight. Rats were administered NOE orally via gavage using a feeding needle. The gavage procedure was performed slowly over no less than 1 min to prevent reflux. NOE treatment began at weaning (PND21) and continued daily for two consecutive weeks. The 14-day window (PND21-34) was selected because PND21 marks weaning and the onset of adolescence in rats, PND21-35 spans an early-to-mid adolescent period of active synaptic pruning and circuit maturation, and MIA-induced neurodevelopmental perturbations remain malleable across this postnatal stage (Meyer, 2019; Coiro et al., 2015). This aligns with established preclinical MIA model standards emphasizing transparent reporting of postnatal intervention timing (Meyer, 2022) and with high-impact adolescent-intervention studies using PND21-onset windows (Reynolds et al., 2024). The PND21-PND35 period overlaps the late-phase refinement window of prefrontal cholinergic circuit maturation and the transition of working memory toward adult-like performance in adolescent rats (Reynolds et al., 2024; Miguelez Fernández et al., 2021; Zoratto et al., 2018; Drzewiecki et al., 2016), representing a tractable window for rescuing MIA-induced neurodevelopmental deficits. A 14-day consecutive dosing paradigm is also adopted in previous studies focusing on natural extract-mediated cholinergic regulation and rodent cognitive phenotypes (Kwon et al., 2025; Lu et al., 2018), supporting the convention of a sustained 14-day course to stably modulate presynaptic cholinergic metabolism and central inflammation, minimizing the risk of acute rebound that typically limits single-dose or ultra-short interventions. The control group received an equivalent volume of saline (using saline as solvent to avoid gastrointestinal irritation from acidic ethanol). All *in vivo* and *ex vivo* experiments were performed on the same cohort of offspring rats at approximately PND 56.

For tissue sample collection and subsequent assays, the IL of each rat was dissected bilaterally: one side of the IL tissue was homogenized and centrifuged to prepare supernatants for the detection of pro-inflammatory cytokines via ELISA; the resulting supernatants were further reused for the quantification of the neurotransmitter ACh, as well as the activity determination of AChE and ChAT. Meanwhile, the contralateral IL tissue was snap-frozen and reserved for Western blot analysis.

Behavioral tests were carried out using two distinct subsets of offspring derived from the same overall experimental cohort: the open field test (OPT) and lever press test were performed on one subset, while the odor delayed non-match-to-sample (DNMS) task was conducted on an independent subset. Notably, *in vivo* single-unit neuronal recordings were performed synchronously during the DNMS task to correlate behavioral performance with IL neuronal firing activity. In addition, *ex vivo* brain slice electrophysiological recordings (including LTP and PPR assays) were also carried out using brain tissues isolated from the same cohort of rats.

Other pharmacological incubation was used only in *ex vivo* slice experiments, with specific conditions detailed in subsequent relevant experimental steps.

### 2.4 Behavioral tests

#### 2.4.1 Open field test

The OFT was used to assess anxiety-like behavior and locomotor activity, as described previously (Sun et al., 2021a; Sun et al., 2022b). The apparatus consisted of a 100 × 100 × 61 cm square arena with black walls and floor, divided into a central zone (80 × 80 cm) and a peripheral zone. Each rat was placed in the center of the arena, and its activity was recorded for 20 min. Primary measures included: total distance traveled, number of entries into the central zone, and time spent in the central zone. Anxiety-like behavior was indicated by reduced entries into and time spent in the central zone.

#### 2.4.2 Lever press test

Following food restriction to 85% of free-feeding weight, rats were trained in sound-attenuated operant chambers (Med Associates) as our previous reports (Sun et al., 2025; Sun et al., 2022c). The chambers contained two retractable levers adjacent to a central food trough, with a cue light above each lever. Delivery of a 45-mg food pellet was paired with an auditory stimulus and illumination of the trough light. Acclimation involved 30-min sessions of non-contingent pellet delivery at 30-s intervals. Upon reliable consumption, daily 30-min training sessions were conducted. In these, a single, randomly selected lever was extended concurrently with its cue light. The schedule of reinforcement was progressively increased through the sequence FR-1, FR-15, FR-30, and FR-60. Advancement required earning 50 pellets per session. Testing on FR-60 continued in 30-min sessions until a criterion of 10 presses per minute was met.

#### 2.4.3 Odor DNMS task

Methods closely followed those described in previous studies (De Falco et al., 2019; Sun et al., 2021b). Briefly, a white plastic cage (90 × 90 × 30 cm^3^) was divided by an opaque Plexiglas divider into equally sized start and test chambers. The testing area was surrounded by a brown curtain to block external visual cues. Odors (0.1 g each of allspice, anise seed, caraway, celery seed, cinnamon, cloves, coffee, cumin, fennel seed, garlic, ginger, mustard powder, nutmeg, oregano, thyme) were evenly mixed into fine sand (80 g). Ceramic bowls (4.0 cm height, 8.5 cm diameter) placed randomly in one of two marked locations along the cage perimeter were used to present the scented sand.

Rats were trained to dig in a bowl of unscented sand to retrieve a buried chocolate chip reward across three phases. In Phase 1, the reward was placed on top of the sand. In Phase 2, it was partially buried. In Phase 3, it was fully buried. Training continued until rats reliably dug for the reward regardless of bowl position. Following dig training, rats were trained on the Non-Match-to-Sample (NMS) task. In the sample phase, a bowl containing one odor (S+) was presented at a random location. After lifting the divider, the rat entered the test chamber. Upon retrieving the chip, the rat was gently guided back to the start chamber for a delay (10 s). In the choice phase, the previously presented bowl (S+) and a second bowl containing a novel odor (S-) were presented at the two possible locations. A correct choice (non-match) was recorded if the rat dug in the bowl with the novel odor (S-). Rats progressed to the DNMS task after achieving the criterion of ≥7 correct choices in eight consecutive trials for three consecutive days during NMS training. Trials in the one-day DNMS task were conducted similarly to NMS trials, except that delay times of 10 s and 30 s were used (Delaney et al., 2024; Kehagia et al., 2010; Xie et al., 2025). Rats were assigned to 10-s and 30-s delay tests in a randomized, group-counterbalanced manner. One week after testing, rats were retrained to the original criterion (≥7/8 correct for 3 consecutive days). The percentage of correct choices in the one-day DNMS task was assessed.

### 2.5 *In vivo* electrophysiological recording

On PND49, rats were anesthetized with sodium pentobarbital (50 mg/kg, i. p.) and secured in a stereotaxic apparatus. The skull was exposed, and a recording electrode was implanted into the IL region of the mPFC (coordinates: 1.5 mm anterior to bregma, 0.5 mm lateral to midline, depth 3.0 mm). The electrode was a tetrode composed of four 12.5 μm diameter tungsten wires, with insulation resistance of 0.5–1.0 MΩ. Animals were given at least 5 days to recover and they were accustomed to the procedure 2 days before the behavioral training and testing.

During the DNMS testing, rats underwent *in vivo* electrophysiological recording. Recording parameters were set as follows: sampling frequency 32 kHz, high-pass filter 600 Hz, low-pass filter 6 kHz. During recording, neuronal firing characteristics were closely monitored, including firing frequency, firing patterns, and temporal relationships to behavioral events. Neuronal spikes were automatically sorted with offline software and KlustaKwik (SpikeSort 3D, Neuralynx) and manually adjusted the clusters using the package of Klusters software to confirm that clusters were distinctly isolated and waveform shapes coincided with action potentials as we reported previously (Sun et al., 2022d; Sun et al., 2021c). Particular attention was paid to delayed-related activity, defined as changes in neuronal firing frequency during the delay period, which is considered a neural correlate of working memory maintenance.

### 2.6 Pharmacological intervention experiments

Pharmacological agents were administered to specifically verify the necessity and sufficiency of cholinergic signaling in NOE-mediated neuroprotection.

To elucidate the mechanism underlying NOE’s regulatory effects on the cholinergic system, a series of pharmacological intervention experiments were designed. AChE inhibitor physostigmine (PHY) was selected to elevate synaptic ACh levels by inhibiting AChE activity. Non-selective muscarinic receptor antagonist scopolamine (SCO) was selected to block M-type cholinergic receptors, inhibiting cholinergic signaling. All drugs were administered via intracerebroventricular (i.c.v.) injection targeting the IL region of the mPFC. A crossover design was employed, with each rat receiving different drug treatments sequentially, with a washout period of at least 48 h between treatments to avoid carry-over effects. Drugs were microinjected precisely into the IL region (0.5 μL/side, based on estimated IL volume to ensure drug localization). The mass doses of approximately 2 μg/side for PHY and 10 μg/side for SCO (Wall et al., 2001; Boix-Trelis et al., 2007; Nunes et al., 2023; Huff et al., 2022). Behavioral testing and electrophysiological recording commenced 30 min post-injection to observe drug effects on performance and neuronal activity.

The differential pharmacological grouping design followed classic gain-of-function and loss-of-function verification paradigms for cholinergic signaling research, which are validated in MIA-induced cognitive deficit models. Briefly, PHY was exclusively administered to the untreated MIA group to verify whether specific enhancement of endogenous cholinergic tone was sufficient to mimic NOE’s therapeutic effects on behavioral and electrophysiological deficits, eliminating the interference of NOE pretreatment. In contrast, SCO was only applied to the MIA+20 mg/kg NOE group (the optimal therapeutic dose confirmed by dose-gradient experiments) to block downstream cholinergic signaling activation, verifying the necessity of functional cholinergic restoration for NOE’s neuroprotective efficacy. Furthermore, consistent with conventional preclinical pharmacological study designs, acute single intracerebroventricular injection of PHY/SCO only induces rapid transient regulation of synaptic cholinergic transmission and neuronal activity for functional phenotyping, but cannot trigger stable alterations in cholinergic metabolic enzyme activity or muscarinic receptor protein expression (Kukla et al., 2026; Sienkiewicz-Jarosz et al., 2003; Udakis et al., 2016). Thus, PHY and SCO intervention groups were not included in subsequent molecular biological analyses, as such acute interventions do not produce detectable molecular-level changes and this arrangement avoids invalid redundant detection in line with peer laboratory standard protocols (Kukla et al., 2026; Sienkiewicz-Jarosz et al., 2003; Udakis et al., 2016).

### 2.7 Molecular biological assays

#### 2.7.1 ELISA measurement of AChE activity, ChAT activity, and ACh content

AChE activity (Wuhan Huamei Cusabio Biotech Co., China. cat. NoCSB-E11304r), ChAT activity (Nanjing Jiancheng Bioengineering Institute, China. cat. No20080721), and ACh content (Abcam. cat. Noab65345) were determined using ELISA kits according to the manufacturer’s instructions. Briefly, tissue was homogenized and centrifuged. The supernatant was reacted with specific substrate/reagents from the respective kits. Absorbance was measured at specified wavelengths, and the concentration/activity of each indicator was calculated based on standard curves.

#### 2.7.2 Western blot analysis of M1/M3 receptor protein expression

IL brain tissue was isolated on ice and lysed in RIPA buffer containing protease/phosphatase inhibitors. After centrifugation, total protein was collected, and concentration was determined using the BCA method, followed by normalization. 50 μg of denatured protein per sample was separated by 10% SDS-PAGE and transferred to PVDF membranes via wet transfer. Membranes were blocked with 5% non-fat milk for 1 h at room temperature, then incubated overnight at 4 °C with primary antibodies: rabbit polyclonal anti-M1 antibody (Alomone Labs. cat. NoAMR-001) at 1:1000 dilution, and rabbit polyclonal anti-M3 antibody (Alomone Labs. Ca. NoAMR-006) at 1:800 dilution. Following thorough washing with TBST, membranes were incubated with HRP-conjugated secondary antibodies, including a 1:10,000 dilution of mouse monoclonal anti-β-actin (Sigma-Aldrich Co. cat. NoA 1978) for 1 h at room temperature. Protein bands were visualized using ECL chemiluminescence. Band intensity was analyzed using ImageJ software. The relative expression levels of M1 and M3 receptors were calculated using β-actin as an internal control. Experiments were repeated three times for validation.

### 2.8 *Ex vivo* brain slice field potential recording and LTP induction

For *ex vivo* recording of LTP in the IL-mPFC, offspring rats were anesthetized, decapitated rapidly, and the brains were dissected into ice-cold, oxygenated (95% O_2_ + 5% CO_2_) artificial cerebrospinal fluid (ACSF), followed by coronal sectioning into 300–400 μm-thick brain slices containing the IL region, which were incubated at 32 °C for 1–2 h to restore viability. Subsequently, the slices were transferred to a recording chamber perfused with oxygenated ACSF (2–3 mL/min, 32 °C), a bipolar tungsten stimulation electrode was placed on the IL afferent fiber pathway, a glass microelectrode (filled with 3 M NaCl, 2–5 MΩ) was inserted into the IL pyramidal cell layer to record field excitatory postsynaptic potentials (fEPSPs). To determine the optimal test stimulation intensity, input-output (I/O) curves were constructed by plotting fEPSP slopes against increasing stimulation intensities, and a test stimulation (0.05 Hz, 0.1 ms pulse width) that evoked 40%–60% of the maximal fEPSP amplitude was applied for 20–30 min to establish a stable baseline. Prior to LTP induction, PPR was measured to assess presynaptic neurotransmitter release probability; pairs of stimuli with inter-stimulation intervals (ISIs) of 15, 30, 60, 120, and 240 ms were delivered, and PPR was calculated as the ratio of the slope of the second fEPSP to that of the first fEPSP. LTP was then induced using theta-burst stimulation (TBS: 10 bursts of 4 pulses at 100 Hz, 200 ms inter-burst interval); after induction, fEPSPs were recorded continuously for 60 min, with LTP defined as a ≥20% increase in fEPSP slope relative to baseline at 60 min post-induction. For drug intervention experiments, brain slices were pre-incubated with PHY (10 μM) or SCO (50 μM) for 30 min prior to baseline recording (Loften et al., 2021; Mei et al., 2020), to assess their effects on LTP induction as well as alterations in I/O curves and PPR across all ISIs.

### 2.9 Statistical analysis

All data are expressed as mean ± standard deviation (Mean ± SD). Behavioral data were analyzed using Repeated Measures ANOVA. Inter-group comparisons were performed using one-way ANOVA, followed by Tukey’s HSD test for post-hoc multiple comparisons. All statistical analyses were performed using SPSS software (edition 21.0). A *P*-value <0.05 was considered statistically significant.

## 3 Results

### 3.1 NOE attenuates MIA-induced neuroinflammation in the IL-mPFC of offspring rats

**FIGURE 1:**
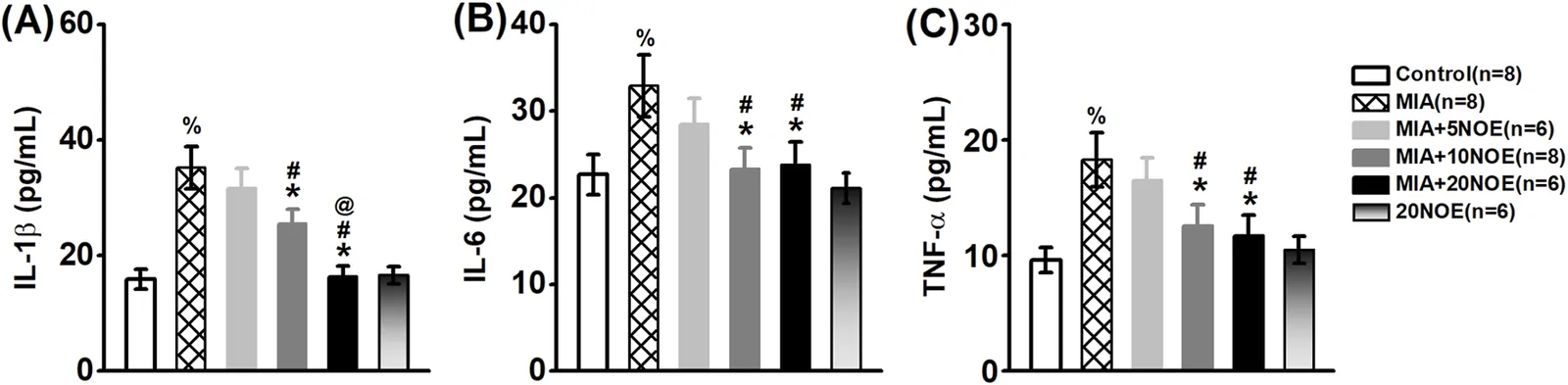
NOE attenuates MIA-induced neuroinflammation in the IL-mPFC of offspring rats. **(A–C)** Bar graphs show the concentrations of pro-inflammatory cytokines. **(A)** IL-1β, **(B)** IL-6, and **(C)** TNF-α in IL-mPFC tissue homogenates from the adolescent offspring of different experimental groups. Statistical significance: Data are presented as mean ± SD. % *P* < 0.05 vs. Control; *P < 0.05 vs. MIA; #*P* < 0.05 vs. MIA+5NOE; @ *P* < 0.05 vs. MIA+10NOE (assessed by one-way ANOVA with post-hoc Tukey’s test).

To determine whether NOE influenced the activity of inflammatory cytokines in the prefrontal cortex of MIA offspring, the poly (I:C)-induced MIA model was employed. ELISA analysis revealed that levels of IL-1β (Figure 1A, one-way ANOVA, *F* (Conway and Brown, 2019; Tsimpili and Zoidis, 2025) = 75.32, *P* < 0.001; MIA vs. Control, *P* < 0.001), IL-6 (Figure 1B, one-way ANOVA, *F* (Conway and Brown, 2019; Tsimpili and Zoidis, 2025) = 19.96, *P* < 0.001; MIA vs. Control, *P* < 0.001), and TNF-α (Figure 1C, one-way ANOVA, *F* (Conway and Brown, 2019; Tsimpili and Zoidis, 2025) = 28.41, *P* < 0.001; MIA vs. Control, *P* < 0.001) were significantly elevated in the IL-mPFC region of MIA offspring compared to controls. NOE treatment dose-dependently decreased these enhanced cytokine levels. Specifically, the 10 mg/kg and 20 mg/kg NOE doses exhibited significant anti-inflammatory effects compared to the MIA group (IL-1β: vs. MIA, both *P* < 0.001; IL-6: vs. MIA, both *P* < 0.001; TNF-α: vs. MIA, both *P* < 0.001), and both were superior to the 5 mg/kg NOE treatment (IL-1β: vs. MIA+5NOE, both *P* < 0.001; IL-6: vs. MIA+5NOE, both *P* < 0.05; TNF-α: vs. MIA+5NOE, both *P* < 0.01). Although the 5 mg/kg NOE dose also attenuated cytokine overexpression to a certain extent, the difference relative to the MIA group failed to reach statistical significance. Furthermore, the 20 mg/kg dose demonstrated superior efficacy in attenuating IL-1β levels compared to the 10 mg/kg dose (MIA+20NOE vs. MIA+10NOE, *P* < 0.001). Treatment with 20 mg/kg NOE did not affect the expression of IL-1β, IL-6, or TNF-α in control offspring (all *P* > 0.05).

### 3.2 NOE modulates cholinergic markers but not muscarinic receptor expression in the IL-mPFC of MIA offspring

**FIGURE 2:**
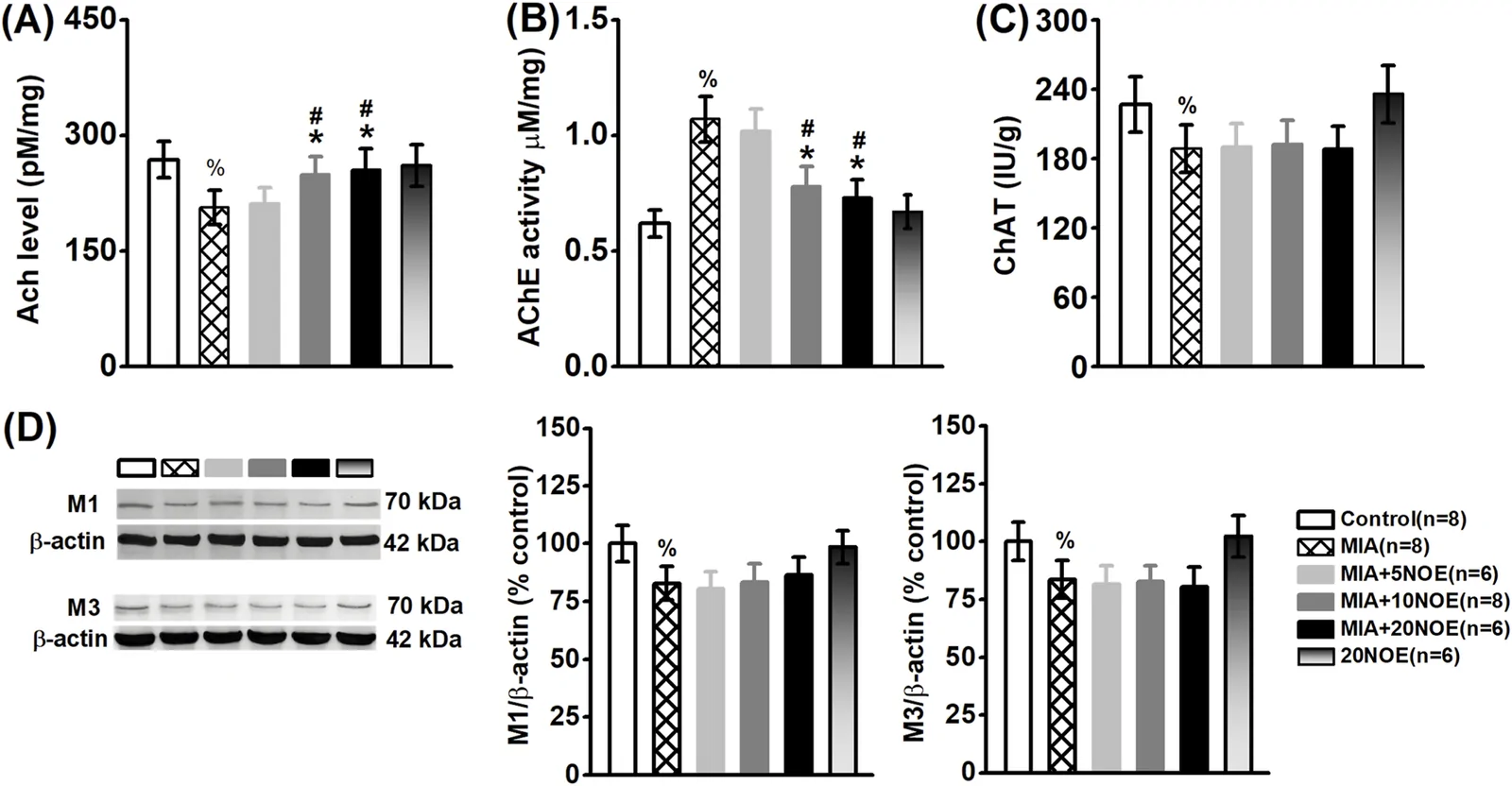
NOE modulates cholinergic markers in the IL-mPFC of MIA offspring. **(A–C)** Effects of NOE on ACh metabolism in the IL-mPFC. Bar graphs show **(A)** ACh content (pM/mg tissue), **(B)** AChE activity (μM/mg tissue), and **(C)** ChAT activity (IU/g tissue) (mean ± SD). **(D)** Effects of NOE on muscarinic acetylcholine receptor expression. (**D**-Left) Representative Western blot images showing protein levels of M1 and M3 muscarinic receptors, with β-actin as the loading control. Right: Quantitative analysis of (**D**-Middle) M1/β-actin and (**D**-Right) M3/β-actin relative protein expression (% of Control). Statistical Significance: Data are presented as mean ± SD. % *P* < 0.05 vs. Control; *P < 0.05 vs. MIA; #*P* < 0.05 vs. MIA+5NOE (assessed by one-way ANOVA with post-hoc Tukey’s test).

Significant differences were observed in the levels of ACh (Figure 2A, one-way ANOVA, *F* (Conway and Brown, 2019; Tsimpili and Zoidis, 2025) = 8.71, *P* < 0.001; MIA vs. Control, *P* < 0.001) and the activity of AChE (Figure 2B, one-way ANOVA, *F* (Conway and Brown, 2019; Tsimpili and Zoidis, 2025) = 36.88, *P* < 0.001; MIA vs. Control, *P* < 0.001) and ChAT activity (Figure 2C, one-way ANOVA, *F* (Conway and Brown, 2019; Tsimpili and Zoidis, 2025) = 6.75, *P* < 0.001; MIA vs. Control, *P* = 0.013) in the IL-mPFC between the control and MIA groups. Following NOE treatment, the ACh level and AChE activity in the IL region were ameliorated in the 10 mg/kg (ACh: MIA+10NOE vs. MIA, *P* = 0.002; AChE: MIA+10NOE vs. MIA, *P* < 0.001) and 20 mg/kg dose groups (ACh: vs. MIA+20NOE, *P* = 0.002; AChE: MIA+20NOE vs. MIA, *P* < 0.001). Compared to the 5 mg/kg NOE treatment, both the 10 mg/kg (ACh: MIA+10NOE vs. MIA+5NOE, *P* = 0.044; AChE: MIA+10NOE vs. MIA+5NOE, *P* < 0.001) and 20 mg/kg (ACh: MIA+20NOE vs. MIA+5NOE, *P* = 0.035; AChE: MIA+20NOE vs. MIA+5NOE, *P* < 0.001) doses were significantly more effective in increasing ACh content and decreasing AChE activity in MIA offspring. The activity of ChAT was declined in MIA group, but no significant effect was found in the three NOE treatment groups (vs. MIA, all *P* > 0.05). Although MIA offspring displayed significantly reduced protein expression of both M1 (Figure 2D-left; Figure 2D-middle, one-way ANOVA, *F* (Conway and Brown, 2019; Tsimpili and Zoidis, 2025) = 8.77, *P* < 0.001; MIA vs. Control, *P* < 0.001) and M3 receptors (Figure 2D-right, one-way ANOVA, *F* (Conway and Brown, 2019; Tsimpili and Zoidis, 2025) = 10.32, *P* < 0.001; MIA vs. Control, *P* < 0.003), NOE treatment at the administered doses did not produce a statistically significant effect on the expression levels of either receptor subtype.

### 3.3 NOE improves working memory deficits in MIA offspring, an effect modulated by cholinergic signaling

**FIGURE 3:**
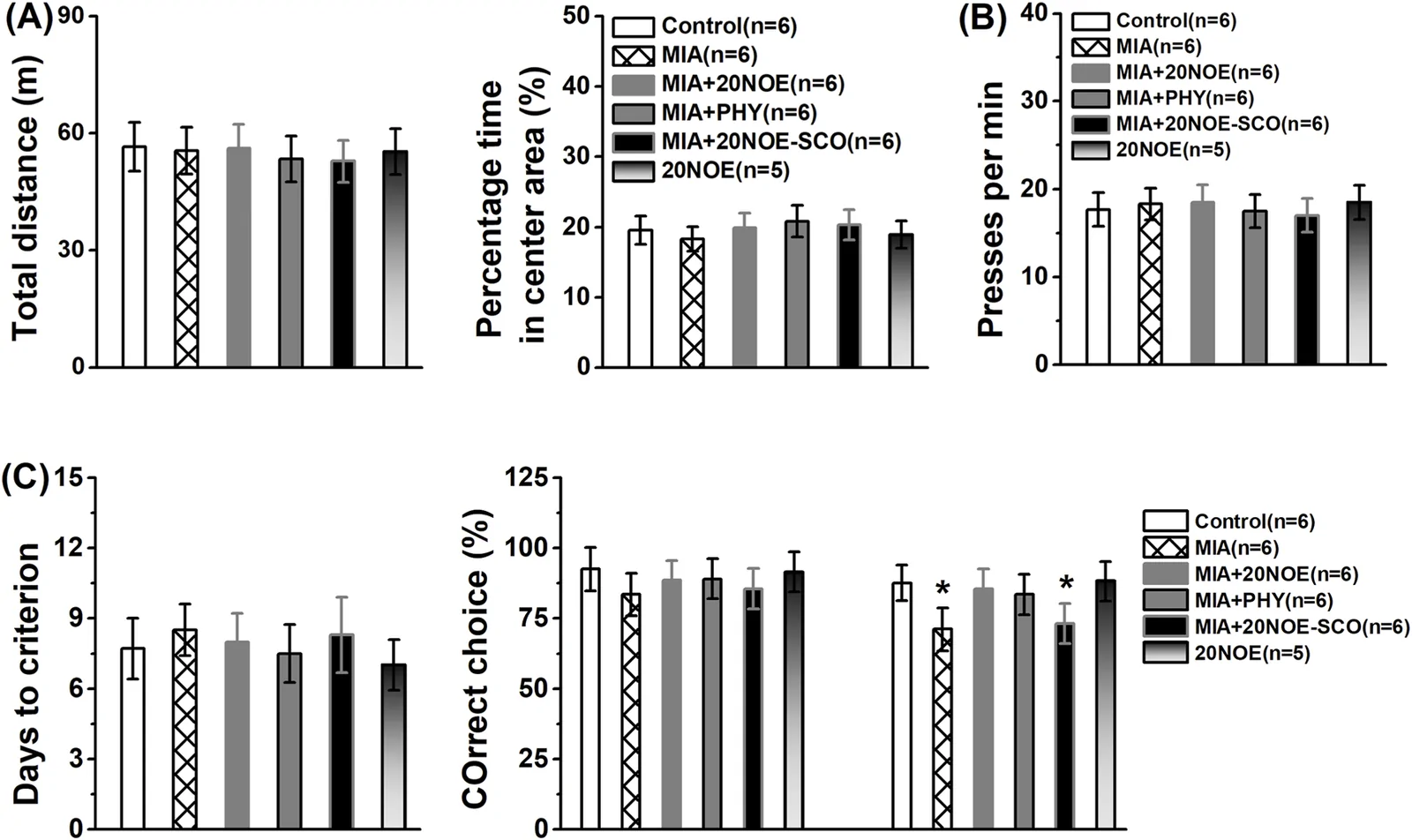
NOE alleviates MIA-induced cognitive deficits via a cholinergic-dependent mechanism. **(A)** Assessment of locomotor activity and anxiety-like behavior in the OFT. (**A**-left) Total distance travelled (m) and (**A**-right) Percentage of time spent in the center area (%) were not significantly altered by MIA or any pharmacological treatment. **(B)** Assessment of motivate behavior in lever press task. Press time was not significantly altered by MIA or any pharmacological treatment. **(C)** Assessment of working memory in the DNMS task. (**C**-left) Days required to reach the pre-set performance criterion in the training stage. No significant differences were observed among the groups. (**C**-right) Percentage of correct choices at 10-s and 30-s delay intervals. MIA offspring exhibited a significant deficit specifically at the 30-s delay. This deficit was significantly ameliorated by NOE (20 mg/kg) or PHY treatment. The cognitive improvement induced by NOE was prevented by the co-administration of SCO. Statistical Significance: Data are presented as mean ± SD. *P < 0.05 vs. Control; (assessed by one-way ANOVA followed by Tukey’s post-hoc test).

The 20 mg/kg NOE dose was selected to investigate the underlying mechanisms in subsequent experiments. As shown in Figure 3A, the total travel distance (Figure 3A-left, one-way ANOVA, *F* (Conway and Brown, 2019; Luo and Kiss, 2021) = 0.39, *P* = 0.849) and the percentage of time spent in the central area (Figure 3A-right, one-way ANOVA, *F* (Conway and Brown, 2019; Luo and Kiss, 2021) = 1.21, *P* = 0.332) in the open field were compared among groups. No statistical difference in lever press time during the lever press task was found (Figure 3B, one-way ANOVA, *F* (Conway and Brown, 2019; Luo and Kiss, 2021) = 0.21, *P* = 0.956). During the training stage of the DNMS olfactory discrimination task, the number of days to reach the performance criterion was similar across groups (Figure 3C-right, one-way ANOVA, *F* (Conway and Brown, 2019; Zheng et al., 2023) = 0.60, *P* = 0.698). In the subsequent discrimination test, the percentage of correct choices was significantly lower in MIA offspring compared to controls when the delay period was increased to 30 s (Figure 3C-left, one-way ANOVA, *F* (Conway and Brown, 2019; Zheng et al., 2023) = 7.15, *P* < 0.001; MIA vs. Control, *P* = 0.002), but not at the 10-s delay (Figure 3C-left, one-way ANOVA, *F* (Conway and Brown, 2019; Zheng et al., 2023) = 1.49, *P* = 0.219). Treatment with 20 mg/kg NOE significantly increased the correct choice percentage at the 30-s delay (MIA+20NOE vs. MIA, *P* = 0.003). However, this improvement was diminished by blocking muscarinic receptors with SCO (MIA+20NOE-SCO vs. MIA+20NOE, *P* = 0.034). Conversely, inhibiting AChE activity with PHY treatment effectively rescued the decreased correct choice percentage (MIA + PHY vs. MIA, *P* = 0.032), demonstrating an improvement comparable to that of 20 mg/kg NOE treatment in MIA offspring (MIA + PHY vs. MIA+20NOE, *P* = 0.994).

### 3.4 NOE rescues synaptic and neuronal activity deficits in MIA offspring via cholinergic mechanisms

**FIGURE 4:**
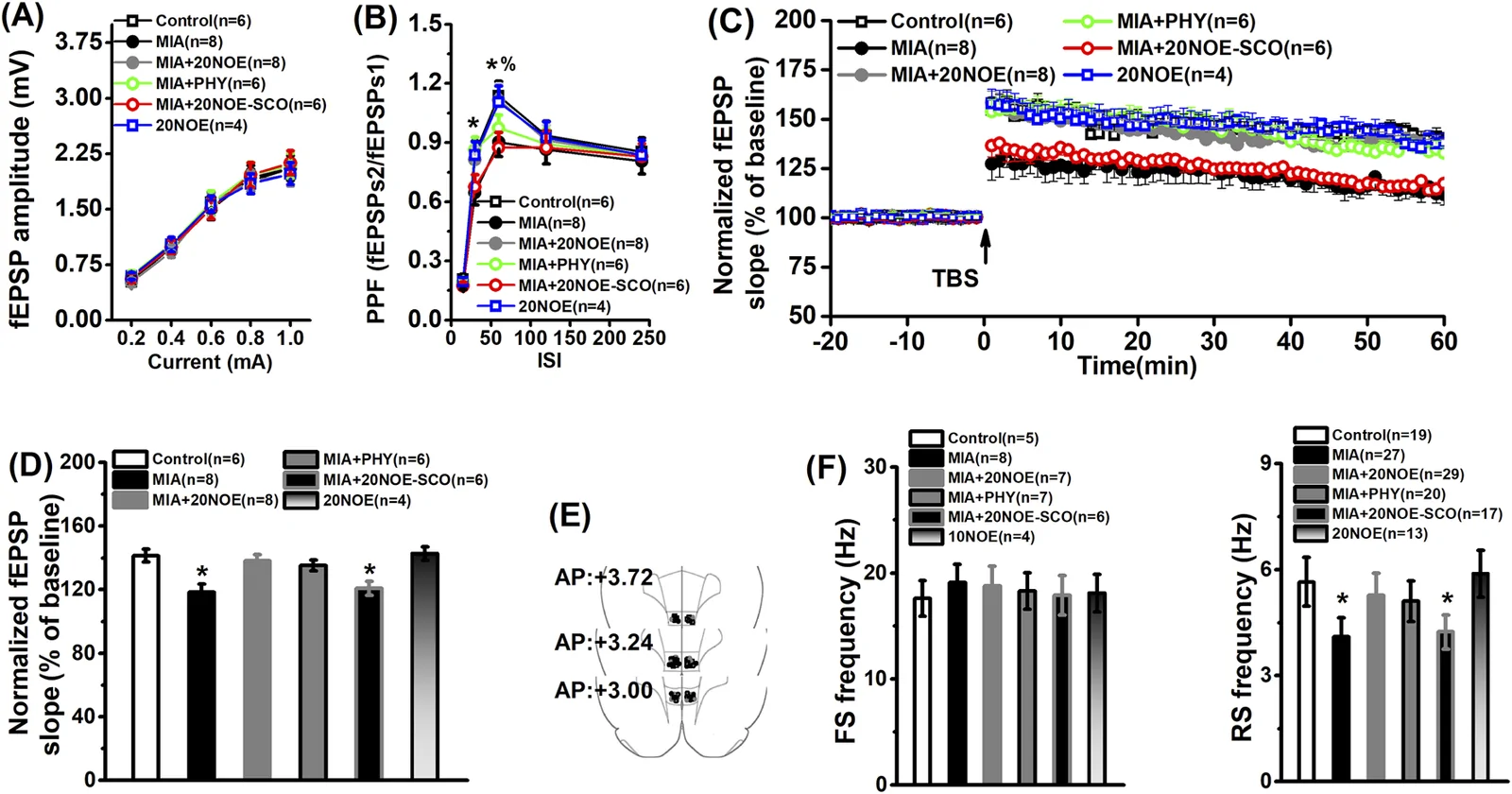
NOE rescues synaptic plasticity and neuronal activity deficits in the IL-mPFC of MIA offspring via cholinergic mechanisms. **(A)** I/O curves plotting the amplitude of fEPSPs against stimulation current intensity show no significant differences in basal synaptic transmission among groups (Repeated-measures ANOVA). **(B)** PPF ratios (fEPSP2/fEPSP1) across different ISIs. MIA offspring exhibit a significant reduction in PPF at 30 ms and 60 ms ISIs. NOE treatment reverses this deficit. This effect is mimicked by PHY at the 30 ms ISI and is blocked by co-administration with SCO. (Repeated-measures ANOVA) **(C)** Time course of fEPSP slope (normalized to baseline) before and after TBS (arrow) for LTP induction. (Repeated-measures ANOVA) **(D)** Quantification of LTP magnitude, expressed as the average normalized fEPSP slope 40–60 min post-TBS. MIA offspring exhibit impaired LTP. NOE treatment rescues this impairment. The rescue effect is comparable to that of PHY treatment and is abolished by SCO co-administration. (one-way ANOVA) **(E)** Schematic representation of coronal rat brain sections indicating the approximate locations of recording electrodes in the IL of mPFC. Anterior-posterior (AP) coordinates relative to bregma are provided. **(F)** *In vivo* single-unit firing frequency analysis. (**F**-Left) Firing frequency of FS interneurons shows no significant differences among groups. (one-way ANOVA) (**F**-Right) Firing frequency of RS (putative pyramidal) neurons during the 30-s delay period of the working memory task. MIA significantly suppresses delay-related firing. NOE treatment restores this activity, an effect that is mimicked by PHY and blocked by SCO. (one-way ANOVA) Statistical Significance: Data are presented as mean ± SD. *P < 0.05, Control, MIA+20NOE and MIA + PHY vs. MIA; %*P* < 0.05 MIA + PHY vs. Control.

In *ex vivo* field potential recordings, no statistical difference was found in I/O curves among groups (Figure 4A, repeated measures of ANOVA, effect of treatment: *F* (Conway and Brown, 2019; Vijayraghavan et al., 2018) = 0.14, *P* = 0.986; interaction effect between treatment and current: *F*_(20, 128)_ = 1.34, *P* = 0.158). The PPR was significantly reduced in MIA offspring at ISIs of 30 ms (Figure 4B, repeated measures of ANOVA, interaction effect between treatment and current: *F*_(20, 128)_ = 4.26, *P <* 0.001; MIA vs. Control, *P* = 0.003) and 60 ms (MIA vs. Control, *P* < 0.001), while NOE treatment effectively reversed this decline at both intervals (MIA+20NOE vs. MIA, 30 ms: *P* = 0.012, 60 ms: *P* < 0.001). Although PHY treatment to some extent facilitated PPR at the 30 ms ISI (MIA + PHY vs. MIA, 30 ms: *P* = 0.023), a significant difference persisted at the 60 ms ISI compared with control group (MIA + PHY vs. Control, 60 ms: *P* = 0.013). Incubation with SCO blocked the beneficial effects of NOE on PPR in MIA offspring slices (MIA+20NOE-SCO vs. MIA+20NOE, 30 ms: *P* = 0.016, 60 ms: *P* = 0.007).

As shown in Figure 4C, robust LTP could be efficiently induced in all groups, and the slopes of fEPSPs stabilized within 5 min following TBS induction (Figure 4C, repeated measures of ANOVA, effect of time: *F*_(59, 1888)_ = 45.73, *P* < 0.001). The fEPSP slope was statistically decreased as a result of the MIA procedure (Figure 4D, one-way ANOVA, *F* (Conway and Brown, 2019; Vijayraghavan et al., 2018) = 40.83, *P* < 0.001; MIA vs. Control, *P* < 0.001), while NOE treatment significantly mitigated this LTP impairment in MIA offspring (MIA+20NOE vs. MIA, *P* < 0.001). The magnitude of this improvement was comparable to that achieved with PHY treatment (MIA + PHY vs. MIA+20NOE, *P* = 0.798). Meanwhile, SCO effectively suppressed the NOE-mediated increase in fEPSP slope in slices from MIA offspring (MIA+20NOE-SCO vs. MIA+20NOE, *P* < 0.001).

In vivo single-unit recordings, totally, 162 units were sorted (125 pyramidal neurons and 37 interneurons) from 39 rats and the recording sites were presented in Figure 4E. Although no statistical difference in the firing frequency of fast-spiking (FS) interneurons was observed (Figure 4F-left, one-way ANOVA, *F* (Conway and Brown, 2019; Martínez-Murillo and Rodrigo, 2020) = 0.65, *P* = 0.661), the firing rate of regular-spiking (RS, putative pyramidal) neurons was significantly declined during the delay period when rats performed the 30-s delay DNMS discrimination test (Figure 4F-right, one-way ANOVA, *F*_(5, 119)_ = 34.76, *P* < 0.001; MIA vs. Control, *P* < 0.001). NOE treatment significantly enhanced this delay-related activity in MIA offspring (MIA+20NOE vs. MIA, *P* < 0.001), while SCO dramatically hindered this beneficial effect of NOE (MIA+20NOE-SCO vs. MIA+20NOE, *P* < 0.001). A similar enhancement of delay-related activity was also observed when the extra-activity of AChE was inhibited by PHY treatment (MIA + PHY vs. MIA+20NOE, *P* = 0.824).

## 4 Discussion

Our study provides integrated evidence that the acidic 70% ethanol NOE ameliorates MIA-induced cognitive deficits by targeting neuroinflammation and, centrally, by functionally restoring cholinergic signaling within the IL-mPFC. The key findings demonstrate that NOE rescues MIA-associated neuroinflammation (IL-1β, IL-6, TNF-α), normalizes cholinergic metabolic markers (elevates ACh, reduces AChE activity), and rectifies the underlying synaptic plasticity (impaired LTP and PPR) and *in vivo* neuronal coding (deficient delay-related activity of pyramidal neurons) deficits. Crucially, the convergence of pharmacological evidence, whereby the benefits of NOE were mimicked by PHY and blocked by SCO, supports the IL-mPFC cholinergic system as the indispensable mediator of NOE’s therapeutic action.

First, we confirmed the anti-inflammatory property of NOE in the MIA offspring brain, aligning with its documented effects in peripheral models (Li et al., 2017; Li et al., 2025; Zhai et al., 2016). By dampening the pro-inflammatory milieu in the IL-mPFC, a region highly vulnerable to developmental immune insults, NOE likely mitigates a primary driver of synaptic dysfunction and neuronal circuit disruption. This provides a permissive foundation for subsequent functional recovery. Second, the core discovery lies in the precise dissection of cholinergic mechanisms. NOE effectively restored the MIA-induced imbalance of ACh metabolism. Notably, the present pharmacological verification was structured to dissociate the sufficiency and necessity of IL-mPFC cholinergic signaling in mediating NOE’s cognitive benefits. By applying the AChE inhibitor physostigmine exclusively to the untreated MIA group to phenocopy NOE-mediated rescue, and the muscarinic antagonist scopolamine exclusively to the NOE-pretreated MIA group to abolish NOE’s functional improvement, we avoided non-specific drug interference and strongly supports cholinergic transmission as the predominant mechanism governing NOE-induced behavioral recovery, synaptic plasticity restoration, and rescue of delay-related neuronal firing. Acute single-dose i.c.v. pharmacological interventions were intentionally excluded from molecular assays, because such acute manipulations only alter transient synaptic ACh occupancy and real-time signaling efficiency, without inducing stable remodeling of AChE/ChAT enzymatic homeostasis or M1/M3 receptor proteostasis (Kukla et al., 2026; Mattio et al., 1986; Plaschke et al., 2014). The critical advance from our pharmacological experiments is the demonstration that direct enhancement of cholinergic tone via AChE inhibition (PHY) is sufficient to replicate key therapeutic outcomes of NOE that is the improvement in 30-s delay working memory performance, partially restoring paired-pulse facilitation (PPR), fully rescuing LTP magnitude, and enhancing delay-related neuronal firing. This powerful parallel suggests that the elevation of synaptic ACh availability is a sufficient strategy to counteract MIA-induced cognitive-pathophysiology (Maia et al., 2025; Djemil et al., 2023). Conversely, the broad antagonism of muscarinic receptors (SCO) negated the benefits of NOE, indicating that its action is necessary dependent on downstream muscarinic receptor activation. The fact that NOE did not alter the downregulated M1/M3 receptor protein expression further sharpens our mechanistic understanding: its primary mode of action is to potentiate cholinergic signaling efficiency (increasing ACh, decreasing its breakdown) rather than modulating receptor density. This functional enhancement appears adequate to overcome the receptor deficiency, driving the observed recovery of synaptic plasticity and neuronal function.

Consistent with typical cholinergic pathological features of poly (I:C)-induced MIA neurodevelopmental models (Jimenez Naranjo et al., 2019; Wu et al., 2015), our molecular validation confirmed significant downregulation of muscarinic receptor protein expression in the IL-mPFC of MIA offspring, accompanied by disrupted presynaptic ACh metabolism characterized by reduced ACh concentration and excessive AChE activity (King and Plakke, 2024; Jimenez Naranjo et al., 2019; Wu et al., 2015). This dual presynaptic and postsynaptic cholinergic dysfunction verifies the successful modeling of MIA-induced prefrontal cholinergic hypofunction and cognitive impairment, which is consistent with previously reported MIA cholinergic phenotypic characteristics. Importantly, emerging preclinical evidence has demonstrated that MIA-triggered muscarinic receptor downregulation originates from prenatal developmental immune programming, maternal poly (I:C) exposure on GD15 durably compresses PFC M1/M4 binding density into adulthood, an effect reversible only by chronic postnatal reprogramming but not by acute cholinergic occupancy (Plaschke et al., 2014; Jimenez Naranjo et al., 2019), resulting in a stable long-term postsynaptic receptor deficiency that is relatively insensitive to postnatal pharmacological intervention (Plaschke et al., 2014; Giovanoli et al., 2016). The present observation that NOE restored IL-mPFC delay-coding without reversing the partial M1/M3 protein downregulation is consistent with this framework. NOE potentiated presynaptic ACh availability rather than erasing a prenatal-programmed postsynaptic receptor deficit. The fact that NOE improved cholinergic signaling and cognitive function without altering M1/M3 receptor abundance reveals a presynaptic dominant functional potentiation mechanism of cholinergic transmission, which is consistent with multiple established cholinergic modulation paradigms (Sarter, 2015; Parikh and Bangasser, 2020; Colangelo et al., 2019). Accumulating preclinical evidence demonstrates that enhanced synaptic ACh availability and reduced AChE-mediated ACh degradation are sufficient to strengthen muscarinic cholinergic signaling efficiency, rescue synaptic plasticity deficits, and restore delay-dependent neuronal coding, independent of changes in postsynaptic muscarinic receptor expression density (Sarter, 2015; Sugisaki et al., 2011; Hornick et al., 2011; Masuoka et al., 2019). Even under conditions of unchanged or partially downregulated receptor protein levels, elevated synaptic ACh concentration can increase receptor occupancy and prolong receptor-mediated downstream signaling activation, thereby compensating for receptor insufficiency and restoring functional cholinergic output (Masuoka et al., 2019; Volpicelli‐Daley et al., 2003; Howe et al., 2017). In the MIA model, persistent neuroinflammation leads to reduced baseline ACh levels and excessive ACh hydrolysis, resulting in insufficient endogenous cholinergic tone to support working memory maintenance (Zhao et al., 2021; Wu et al., 2015). NOE functionally rescues cholinergic dysfunction primarily by correcting presynaptic ACh metabolic imbalance rather than modulating postsynaptic receptor expression. Notably, our pharmacological verification further supports this mechanism: the AChE inhibitor physostigmine replicated NOE’s beneficial effects on neuronal firing and synaptic plasticity without changing receptor expression, whereas muscarinic receptor blockade completely abolished NOE-mediated functional recovery. This indicates that functional upregulation of cholinergic tone via presynaptic metabolic improvement, rather than postsynaptic receptor upregulation, is the important mechanism underlying NOE’s pro-cognitive action, which is a common and well-validated regulatory pattern in prefrontal cholinergic neuromodulation.

A methodological clarification is warranted regarding the present cholinergic inference. Although NOE elevated synaptic ACh availability and reduced AChE activity, and although local muscarinic antagonism entirely negated its rescue of LTP and delay-period firing, the present study supports cholinergic mediation through convergent functional pharmacologyrather than direct visualization of cholinergic terminals or synapses. We therefore refrain from claiming structural restoration of the cholinergic innervation, and instead emphasize that NOE functionally reinstates cholinergic tone sufficient to normalize IL-mPFC circuit output during working memory maintenance.

**FIGURE 5:**
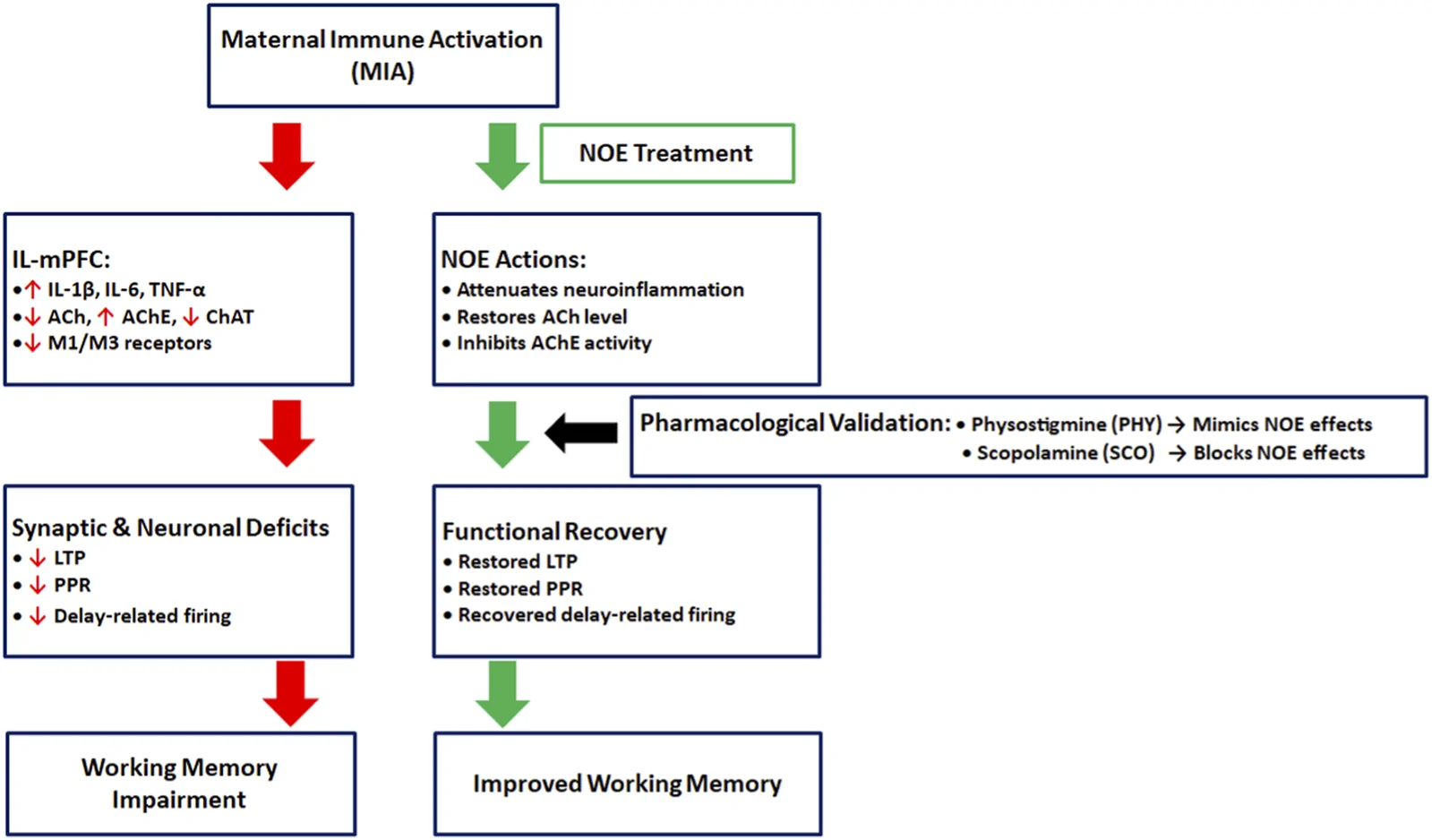
Schematic illustration summarizing the proposed mechanism linking NOE, neuroinflammation, cholinergic signaling, synaptic plasticity, neuronal firing, and working memory improvement. Maternal immune activation (MIA) induces elevated pro-inflammatory cytokines (IL-1β, IL-6, TNF-α), reduced acetylcholine (ACh) and choline acetyltransferase (ChAT), and increased acetylcholinesterase (AChE) activity within the infralimbic medial prefrontal cortex (IL-mPFC) of adolescent rat offspring, while MIA-induced downregulation of M1/M3 muscarinic receptor expression cannot be reversed by NOE treatment. This cholinergic disturbance causes impaired synaptic plasticity (decreased LTP and PPR) and attenuated delay-related firing of pyramidal neurons, ultimately leading to working memory impairment. Treatment with *Nauclea officinalis* extract (NOE) attenuates neuroinflammation, restores ACh levels and inhibits AChE activity, without reversing MIA-induced ChAT reduction or changing M1/M3 receptor expression. These protective effects trigger functional recovery of synaptic plasticity and neuronal activity, resulting in improved working memory. Pharmacological validation demonstrates that physostigmine (PHY) mimics NOE-mediated beneficial outcomes, whereas scopolamine (SCO) abolishes all therapeutic effects of NOE.

Our findings support the established link between IL-mPFC cholinergic hypofunction and working memory deficits in schizophrenia models (Maia et al., 2025; Vijayraghavan et al., 2018; Wall et al., 2001). The novelty of this work is multifaceted. To our knowledge, this is the first study to demonstrate the therapeutic efficacy of NOE against the spectrum of behavioral, synaptic, and *in vivo* neuronal deficits in an MIA neurodevelopmental model.​ More importantly, we move beyond correlation to suggest causality and sufficiency within the cholinergic pathway. By showing that PHY alone can mimic NOE’s effects on LTP and delay-period firing, we provide direct evidence that rectifying cholinergic deficiency is a central, effective therapeutic strategy. However, the observation that NOE’s effect on short-term plasticity (PPR) was more complete than that of PHY suggests potential additional, synergistic mechanisms of the extract. This may be rooted in its anti-inflammatory action, which could optimize presynaptic function beyond mere elevation of ACh (Halder and Lal, 2021; Reale and Costantini, 2021). Furthermore, emerging evidence has confirmed that MIA-induced prefrontal cognitive deficits are also governed by glutamatergic and dopaminergic neurotransmission disorders (Maddock et al., 2024; Del Olmo et al., 2026; Weber-Stadlbauer et al., 2021). As a multi-component natural extract, NOE may indirectly regulate these neurotransmitter systems via anti-inflammatory or neuromodulatory effects, thereby synergistically improving working memory function (Liu et al., 2022; Li et al., 2023; Zhai et al., 2016). Collectively, these findings position NOE not merely as a cholinergic enhancer, but as a multi-target therapeutic agent that may offer broader circuit normalization than pure AChE inhibition, with IL-mPFC cholinergic signaling as its primary functional target. Together, these observations support a unifying mechanistic model. As diagrammatically integrated in Figure 5, the presynaptic-dominant, receptor-density-independent potentiation of IL-mPFC cholinergic signaling constitutes the central interpretive framework for the present dataset.

Notably, rather than employing a battery of spatial working memory assays, the present study adopted the olfactory DNMS delay task as the primary and unified behavioral readout. Spatial tasks such as the Morris Water Maze or Y-maze rely extensively on dorsal hippocampal circuit computations, whereas the olfactory DNMS delay task is a well-established mPFC-dependent working memory paradigm in rodents (De Falco et al., 2019; Benoit et al., 2020). Previous MIA studies have similarly favored mPFC-dissociable assays, such as attentional set shifting and object-location memory, to minimize off-target hippocampal interference when interrogating prefrontal pathophysiology (Woods et al., 2026; Woods et al., 2023; Maleninska et al., 2022; Canetta et al., 2016). By aligning our behavioral readout with this precedent, we ensured that all downstream validations (*in vivo* mPFC neuronal firing, intra-mPFC synaptic plasticity, and local pharmacological intervention) interrogated the exact same circuit-behavior axis. The convergent rescue at the electrophysiological, synaptic, and pharmacological levels supports the reliability of this single-paradigm design, indicating that NOE exerts specific, mPFC-mediated working memory benefits rather than a broad, non-specific cognitive enhancement.

Several limitations should be considered. First, our mechanistic resolution is presently pharmacological rather than anatomical. While convergent local scopolamine blockade and AChE inhibition functionally isolated the cholinergic axis across behavioral, synaptic, and *in vivo* neuronal levels. We acknowledge that future pharmacological studies incorporating M1/M4-subtype selective dissection, alongside anatomical terminal mapping, will help consolidate the cellular substrates identified here. Second, NOE is a complex alkaloid mixture. Consistent with conventional research limitations of natural plant extracts in current pharmacological studies, the present work only verified the overall neuroprotective efficacy of total NOE, without conducting bioactivity-guided fractionation and isolated screening of individual active constituents. Although strictosamide has been confirmed as the dominant bioactive monomer of NOE by numerous preclinical studies, the independent functional contribution of strictosamide and synergistic mechanisms among different alkaloid components in improving MIA-induced cholinergic dysfunction and working memory deficits still require systematic verification. Future studies will focus on bioactivity-guided component separation, *in vitro* and *in vivo* activity verification of single alkaloid monomers, and clarification of the synergistic pharmacological network among multiple constituents to further improve the translational value of this research. Third, our electrophysiological recordings were confined to the IL-mPFC. Examining how NOE-induced local changes modify functional connectivity within prefrontal-hippocampal-thalamic networks would provide a systems-level understanding of cognitive improvement. Fourth, the present study was restricted to male adolescent offspring. While this design minimized hormonal variance during our initial mechanistic isolation of IL-mPFC cholinergic signaling, MIA is known to produce sexually dimorphic neuroimmune and cognitive phenotypes (Sal-Sarria et al., 2024; Meehan et al., 2016; Harbi and Mouihate, 2025). Future studies must systematically incorporate female cohorts to determine whether NOE’s anti-inflammatory and cholinergic benefits generalize across sexes or interact with estrous-cycle-dependent circuit modulation. Additionally, our interventions and recordings were confined to the peri-adolescent window. Consequently, the present data confirm acute NOE-mediated rescue of adolescent working memory circuits, but do not establish whether these benefits persist once animals transition into adulthood. Longitudinal designs tracking the same subjects from adolescence into early adulthood will be essential to clarify whether peri-adolescent NOE administration produces enduring synaptic reprogramming or requires sustained dosing. Finally, the single olfactory DNMS behavioral paradigm is a limitation of the present study, and multi-task cognitive verification will be adopted in future research to confirm the generalizability of NOE’s cognitive protective effects.

In conclusion, this study demonstrates that NOE significantly alleviates working memory deficits and associated neuropathophysiology in a MIA model. Its efficacy is mediated through a dual mechanism encompassing the attenuation of neuroinflammation and, decisively, the functional restoration of cholinergic neurotransmission in the IL. The compelling evidence that direct AChE inhibition replicates core therapeutic benefits of NOE underscores the centrality of cholinergic potentiation as a viable treatment strategy. Conversely, the blockade of these benefits by muscarinic antagonism confirms the necessity of this pathway. These findings not only identify NOE as a promising multi-target candidate for schizophrenia-related cognitive impairments but also strongly support the IL-mPFC cholinergic system as a critical and efficacious target for intervention, paving the way for novel therapeutic development.

## Data Availability

The datasets presented in this study can be found in online repositories. The names of the repository/repositories and accession number(s) can be found below: https://figshare.com/, https://doi.org/10.6084/m9.figshare.32780775.
